# Glycerol kinase-like proteins cooperate with Pld6 in regulating sperm mitochondrial sheath formation and male fertility

**DOI:** 10.1038/celldisc.2017.30

**Published:** 2017-08-22

**Authors:** Yuxi Chen, Puping Liang, Yan Huang, Minyan Li, Xiya Zhang, Chenhui Ding, Junyan Feng, Zhen Zhang, Xueqing Zhang, Yuanzhu Gao, Qinfeng Zhang, Shanbo Cao, Haiyan Zheng, Dan Liu, Zhou Songyang, Junjiu Huang

**Affiliations:** 1 Key Laboratory of Gene Engineering of the Ministry of Education, Institute of Healthy Aging Research and State Key Laboratory of Biocontrol, SYSU-BCM Joint Research Center, School of Life Sciences, Sun Yat-sen University, Guangzhou, China; 2 State Key Laboratory of Ophthalmology, Zhongshan Ophthalmic Center, Sun Yat-sen University, Guangzhou, China; 3 Key Laboratory of Reproductive Medicine of Guangdong Province, School of Life Sciences and the First Affiliated Hospital, Sun Yat-sen University, Guangzhou, China; 4 Key Laboratory of Reproductive Medicine of Guangdong Province, Third Affiliated Hospital of Guangzhou Medical University, Guangzhou, China; 5 Cell-Based Assay Screening Core, Verna and Marrs Mclean Department of Biochemistry and Molecular Biology, Baylor College of Medicine, One Baylor Plaza, Houston, TX, USA

## Abstract

Spermatids undergo the final steps of maturation during spermiogenesis, a process that necessitates extensive rearrangement of organelles such as the mitochondria. Male infertility has been linked to mitochondrial disorder, for example, hypospermatogenesis and asthenozoospermia. However, the mechanisms that regulate mitochondrial dynamics during spermiogenesis remain largely unknown. We found the glycerol kinase (Gyk)-like proteins glycerol kinase-like 1 (Gykl1) and glycerol kinase 2 (Gk2) were specifically localized to the mitochondria in spermatids. Male mice deficient in either *Gykl1* or *Gk2* were infertile due to dysfunctional spermatozoa, which exhibited unregulated ATP production, disordered mitochondrial sheath formation, abnormal mitochondrial morphology, and defective sperm tail. We demonstrated that the unique C-terminal sequences found in Gykl1 and Gk2 mediated their targeting to the mitochondrial outer membrane. Furthermore, both Gykl1 and Gk2 could interact with Pld6 (MitoPLD) and induce Pld6 and phosphatidic acid (PA)-dependent mitochondrial clustering in cells. Taken together, our study has revealed previously unsuspected functions of Gyk-like proteins in spermiogenesis, providing new insight into the potential mechanisms that lead to spermatozoa dysfunction and male infertility.

## Introduction

Mitochondria are sites of oxidative phosphorylation and essential for cellular energy production [1]. Mitochondria are highly dynamic and motile. They change in shape and size and undergo fusion, fission, transport and mitophagy, which involve complicated steps and require coordination [2,3,4]. For example, arrangement of mitochondria may depend on their trafficking along the cytoskeleton [5,6,7]. In response to internal and external stimuli, mitochondria undergo constant fusion and fission. Fusion may occur to ensure mitochondrial homogeneity in response to metabolic and environmental stress, and fission can eliminate damaged mitochondria and facilitate apoptosis [2].

While the exact mechanisms and physiological significance of mitochondria dynamics remain under active investigation, multiple players have been uncovered. For instance, both mitochondrial fusion and fission appear to be mediated by mitochondrial membrane proteins and dynamin family members [4, 8,9,10, 11,12,13]. One widely studied regulatory pathway involves lipids such as cardiolipid (CL), phosphatidic acid (PA), diacylglycerol (DAG) and lysophosphatidic acid (LPA) [14, 15]. The observation that phospholipase D family member 6 (Pld6/MitoPLD) induces mitochondrial fusion by generating PA from CL indicates that PA may act as a signaling molecule in controlling mitochondrial dynamics [4]. Further investigations showed that the phosphatidate phosphatase LIPIN1 could terminate mitochondrial fusion by converting PA into DAG [16]. Phosphatidic acid-preferring phospholipase A1 (Pa-pla_1_) can also deacylate PA into LPA, thus inhibiting mitochondrial fusion induced by Pld6 [1].

Normal mitochondrial function and dynamics are intimately linked to biological processes such as cell growth, proliferation and differentiation. For instance, the number, shape, structure and dynamics of mitochondria change dramatically during mammalian spermatogenesis. Secondary spermatocytes, spermatids, and sperms have more condensed forms of mitochondria compared with spermatogonia and primary spermatocytes [17]. During spermiogenesis, the mitochondria undergo reduction and rearrangement and form tubular structures that make up the sperm midpiece [18, 19]. The mitochondrial sheath is tightly packed in the midpiece of the sperm flagellum and is thought to be responsible for generating the ATP required for sperm motility [7, 20]. The evolutionarily conserved mitochondrial sheath structure, which is important for spermatozoa motility, underlines the distinct and important function of mitochondria in spermatogenesis [17, 19, 21]. A number of proteins have been reported to be involved in mitochondrial dynamics during spermatogenesis, including the PTEN-induced putative kinase 1 (PINK1), Pld6 and Pa-pla_1 _[1, 4, 8, 16, 22]. Male infertility has been linked to mitochondrial disorders such as hypospermatogenesis with abnormal lower mtHSP60 immunoreactivity and asthenozoospermia with mutation of the *CO*_III_ gene [23, 24]. Collectively, these studies demonstrate that tight regulation of mitochondrial function is essential for proper spermatogenesis. However, the regulatory process that modulates the mitochondrial sheath formation during spermiogenesis remains to be identified.

We report here the identification of glycerol kinase (Gyk)-like proteins glycerol kinase-like 1 (Gykl1) and glycerol kinase 2 (Gk2) as new players that specifically regulate sperm mitochondrial sheath formation. Gyk is the enzyme responsible for converting glycerol into glycerol 3-phosphate, the substrate for glycolysis and lipid synthesis [25,26,27, 28]. Two *Gyk-*related genes *Gykl1* and *Gk2* were first identified as *Gyk*-like intronless retroposons and found exclusively expressed in mouse testis [29, 30]. Mouse Gykl1 (549 a.a.) and Gk2 (554 a.a.) are highly similar in protein sequence but differ slightly in molecular weight. Human appears to have only one glycerol kinase (Gyk)-like protein, GK2 [29]. In contrast to Gyk, neither Gykl1 nor Gk2 exhibits detectable glycerol kinase activity *in vitro* [30]. Interestingly, decreased GK2 mRNA expression in males with teratozoospermia and increased GK2 protein levels in asthenozoospermia patients have also been reported [31, 32], suggesting regulated expression of GK2 may be a key factor in male fertility.

We found that Gykl1 and Gk2 were exclusively expressed and localized to mitochondria in round and elongated spermatids and retained on the mitochondrial sheath in spermatozoa. Furthermore, deletion of either *Gykl1* or *Gk2* in mice, by CRISPR/Cas9-mediated gene knockout (KO), impacted mitochondrial morphology and mitochondrial ATP production as well as midpiece and sperm tail integrity in spermatozoa, compromising spermatozoa motility and leading to male infertility. Gykl1 or Gk2 may anchor itself to the mitochondrial outer membrane through its C-terminal mitochondrial localization sequence. Overexpression of Gykl1 or Gk2-induced mitochondrial clustering in a PA-dependent manner. Both Gykl1 and Gk2 can interact with Pld6, possibly serving as activators of Pld6 function. Our data not only shed light on the factors and pathways that regulate mitochondrial dynamics and integrity but also underscore the crucial role played by Gyk-like proteins during spermiogenesis. These findings also have important clinical implications for patients with mitochondria-related reproductive disorders, and for the development of new and effective contraceptives.

## Results

### Mouse *Gykl1 and Gk2* are specifically expressed in round and elongating spermatids

**Figure 1:**
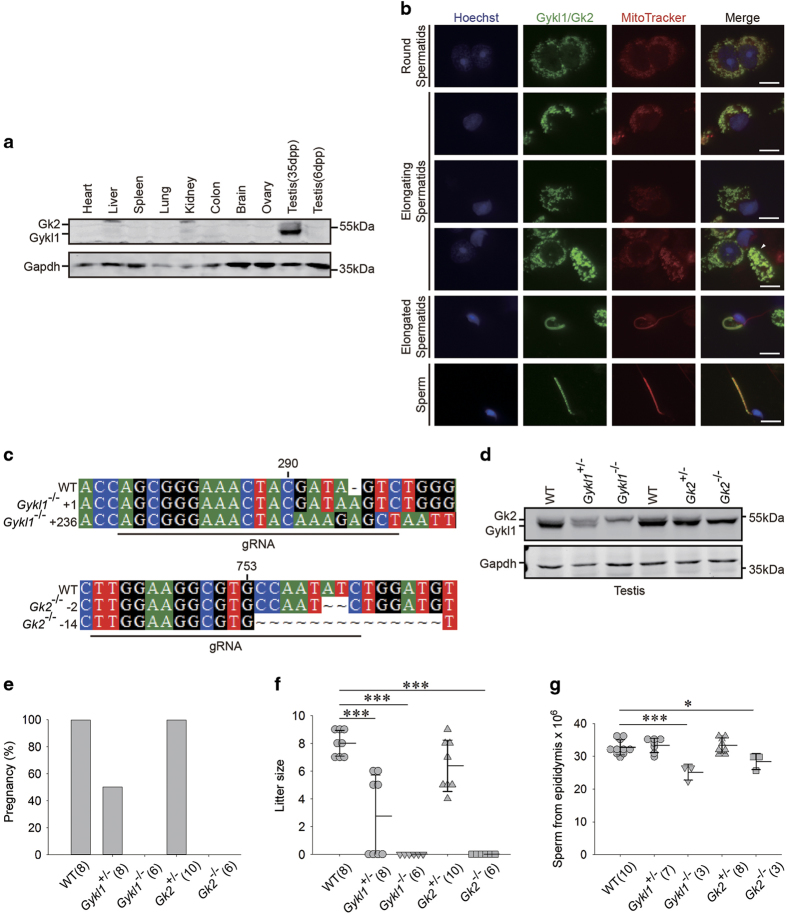
Mice deficient in *Gykl1* or *Gk2* exhibit reproductive defects. (**a**) Immunoblotting of Gykl1 and Gk2 protein in various tissues from C57BL/6J mice. dpp: day post partum. (**b**) Immunostaining of Gykl1 and Gk2 in round spermatids, elongating spermatids and spermatozoa pre-stained with 150 nM MitoTracker Red CMXRos. White arrow indicates the mitochondria in elongating spermatid. Scale bar: 10 μm. (**c**) *Gykl1* and *Gk2* KO mice were generated by CRISPR/Cas9-mediated genome editing. sgRNA sequences that target the *Gykl1* and *Gk2* loci are indicated here. Wild-type (WT) sequences are aligned against sequences from both mutant alleles for each mutant mouse. '*n*' denotes the number of nucleotides inserted or deleted in the region. (**d**) Whole-cell extracts were collected from the testes of wild-type and *Gykl1*/*Gk2* KO mice, and blotted with antibodies against Gykl1/Gk2. An antibody against GAPDH was used as a loading control. (**e**–**g**) The mean pregnancy rate (**e**), litter size (**f**) and sperm count (from the epididymis) (**g**) for wild-type vs homozygous (−/−) and heterozygous (+/−) *Gykl1* and *Gk2*-deficient mice were determined and compared. Error bars represent s.d. Numbers in parentheses indicate sample size (*n*). Significance was determined by ANOVA. *P<0.05. **P<0.01. ***P<0.001.

In a search for specific spermatogenesis-regulatory proteins, we identified mouse Gyk-like proteins Gykl1 and Gk2 as two possible candidates [33]. Gyk is highly conserved across species. Patients with X-linked recessive glycerol kinase deficiency (GKD), which is caused by enzymatic inactivation or *Gyk* deletion [34], suffer from asymptomatic hyperglyceridaemia, growth and psychomotor retardation and metabolic disorders [25, 34, 35]. Knockout of *Gyk* in mice leads to postnatal growth retardation, abnormal fat metabolism, autonomous glucocorticoid synthesis, and early death (within 3–4 days) [25]. Gykl1 and Gk2 are highly similar to Gyk and to each other (Supplementary Figure S1A). However, they exhibit no glycerol kinase activities *in vitro* and their functions remain unknown [30]. Unlike *Gyk*, which was detectable in the kidney and brain of C57BL/6J mice, *Gykl1 and Gk2* mRNAs were specifically found in mouse testis (Supplementary Figure S1B) [29, 33]. To examine the protein expression pattern of Gykl1 and Gk2 in different tissues, we raised a rabbit polyclonal antibody against recombinant full-length Gykl1 protein. The antibody could detect ectopically expressed Gykl1 and Gk2 in HEK293T cells (Supplementary Figure S1C), as well as endogenous Gykl1 and Gk2 proteins in mouse testes (Figure 1a), and henceforth was designated anti-Gykl1/Gk2 antibody. Consistent with their mRNA expression patterns, Gykl1 and Gk2 protein expression appeared to be restricted to mature testes (Figure 1a).

The first wave of mouse spermatozoa appears between 1 and 35 days post partum (dpp), representing the developmental stages of spermatogenesis. During this process, spermatogonia (1–6 dpp) differentiate into primary and secondary spermatocytes (7–14 dpp) and later into round (15–21 dpp) and elongating spermatids (22–35 dpp) [36]. When we examined the expression of Gykl1/Gk2 in lysates of testes from different days post partum, we found strong signals of Gykl1 and Gk2 in 35, 49 and 70 dpp samples, indicative of their expression in spermatids (Supplementary Figure S1D). Immunostaining of seminiferous tubules at different spermatogenesis stages showed intense expression of Gykl1 and Gk2 protein at 21 and 35 dpp, indicating the expression of Gykl1 and Gk2 is as early in round spermatids (Supplementary Figure S1E). To further investigate the specific cell type and developmental stages in which Gykl1 and Gk2 were expressed, testis sections from 35 dpp C57BL/6J mice were stained with the Gykl1/Gk2 antibody along with antibodies against the spermatogonia stem cell marker PLZF and the meiosis marker SYCP3 (Supplementary Figure S1F). Only cells close to the lumen stained positive for Gykl1/Gk2. No overlap of signals was found for Gykl1/Gk2 with either PLZF or SYCP3, supporting the notion that Gykl1 and Gk2 proteins were restricted to round and elongating spermatids and absent from spermatogonia and spermatocytes. We speculated that the punctate and tubular staining patterns of Gykl1 and Gk2 in round and elongating spermatids were suggestive of mitochondrial localization, and proceeded to co-stain round and elongating spermatids from testis and mature spermatozoa from epididymis for Gykl1/Gk2 and MitoTracker. Indeed, we found overlapping signals of Gykl1/Gk2 with MitoTracker (Figure 1b), strongly suggesting that Gykl1 and Gk2 are specifically localized to the mitochondria in round and elongating spermatids.

### Male mice deficient in *Gykl1* or *Gk2* display reproductive defects

To better understand the function of *Gykl1* and *Gk2 in vivo*, we generated mice knocked out for either *Gykl1* or *Gk2* using CRISPR/Cas9 as previously described, and obtained two mutant lines for each gene (Figure 1c) [37,38,39]. Genomic sequencing revealed frame-shift mutations that resulted in premature translation termination in all cases (Figure 1c). Successful disruption of Gykl1 or Gk2 expression was confirmed by Western blotting of protein extracts of the testis using our anti-Gykl1/Gk2 antibody (Figure 1d). Deletion of *Gykl1* did not appear to affect the expression of *Gk2* and vice versa (Figure 1d). We found no gross changes compared with controls in testis morphology and size or germ cell arrangements in the seminiferous tubules in the KO mice (Supplementary Figure S2A–S2C), suggesting normal testis morphogenesis in the mutant mice.

When mated with wild-type C57BL/6J females, *Gykl1*^−/−^ and *Gk2*^*−/−*^ males appeared infertile, and significant reduction (50%) in pregnancy rates was noted for the *Gykl1*^+/−^ mice (Figure 1e). The litter size of *Gykl1*^+/−^ males (2.8±3.0) was significantly smaller than that of WT mice (8.0±1.0) (Figure 1f). Furthermore, fewer spermatozoa were obtained from the epididymis of *Gykl1*^−/−^ and *Gk2*^−/−^ mice (3.28×10^7^ for WT, 2.52×10^7^ for *Gykl1*^−/−^, and 2.84×10^7^ for *Gk2*^−/−^) (Figure 1g). Therefore, *Gykl1*^−/−^ and *Gk2*^*−/−*^ mice clearly display reproductive defects despite apparently normal testis morphogenesis.

### *Gykl1*^*−/−*^ and *Gk2*^*−/−*^ males exhibit mitochondrial defects in the spermatozoa

To probe the causes of infertility and reproductive defects in *Gykl1* and *Gk2* KO mice, we carried out computer-assisted spermatozoa analysis (CASA) to document spermatozoa motility, including curvilinear velocity, straight-line velocity, and amplitude of lateral head movement in the mutant mice. As shown by all the parameters we examined (Table 1; Supplementary Movies S1,Supplementary Movies S2), spermatozoa deficient for *Gykl1* or *Gk2* showed drastically reduced activities.

**Figure 2:**
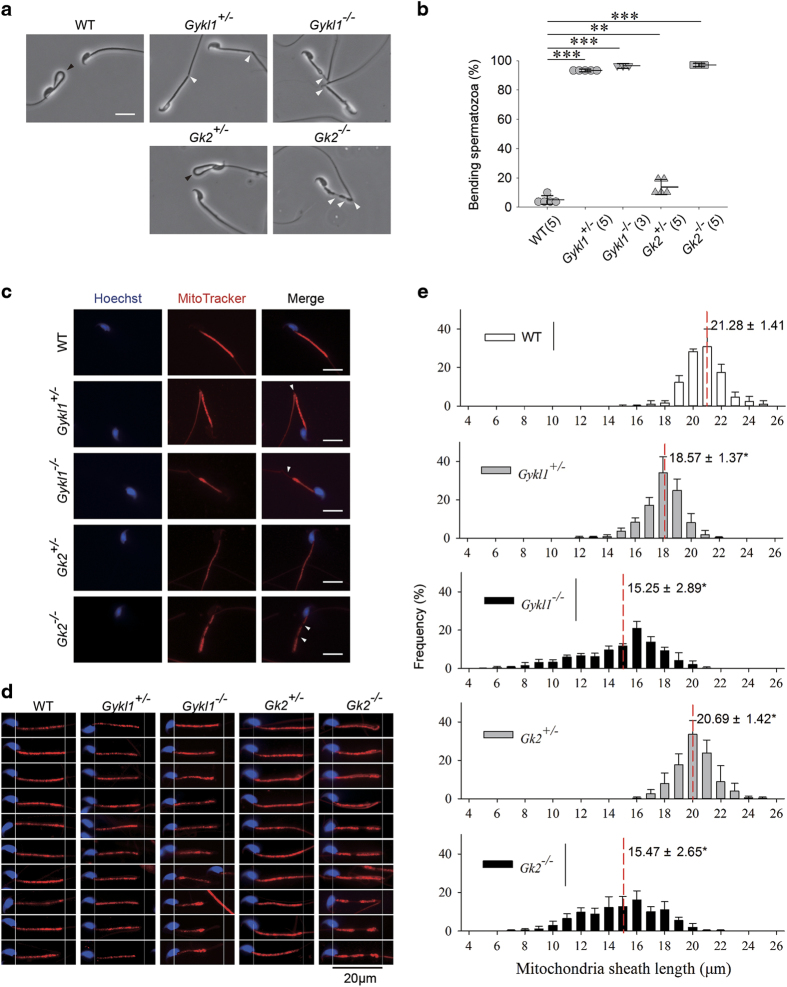
The spermatozoa from male *Gykl1* and *Gk2* KO mice display mitochondrial defects. (**a**) The spermatozoa from the epididymis of wild-type and mutant mice were visualized under the microscope. Black arrows indicate normal bending of sperms which is smooth. White arrows indicate abnormal gaps between the mitochondrial sheath and tail. (**b**) The spermatozoa from (**a**) were scored and plotted to calculate the mean percentage of spermatozoa with bending (e.g., hairpins or gaps). Error bars indicate s.d. Numbers in parentheses indicate sample size (*n*). Significance was determined by the ANOVA. **P<0.01. ***P<0.001. (**c**) The spermatozoa from various mice were stained with MitoTracker dye to visualize the mitochondria and Hoechst 33342 was used to visualize the nuclei. White arrows indicate gaps between the mitochondrial sheath and tail. Scale bar: 10 μm. (**d** and **e**) The spermatozoa from various mice were collected to measure the length of mitochondrial sheath. Some representative spermatids measured in **e** are shown in **d**. The distribution of the measurements is plotted in **e**. The red line indicates average length. Error bars represent s.d. More than 300 spermatozoa were collected from 3–5 animals of each group. Significance was determined by the ANOVA. *P<0.05, compared with WT.

Under microscopes, normal spermatozoa appear relaxed or bent (resembling a smooth string), with the midpiece connected smoothly to the tail (Figure 2a). In *Gykl1*-KO spermatozoa, however, a gap between the midpiece and tail was clearly present due to an abrupt bending that affected its morphology. The same abnormal hairpin-shaped morphology was also found in spermatozoa from *Gykl1*^+/−^ mice. Interestingly, the midpiece of the spermatozoa from *Gk2*^−/−^ mice was split into several pieces (Figure 2a). The percentage of abnormally shaped spermatozoa was significantly high in mice carrying homozygous or heterozygous deficient for *Gykl1* (^+/−^: 93.1±0.9%, ^−/−^: 96.3±1.6%) or *Gk2* (^+/−^: 13.9±5.2%, ^−/−^: 96.9±1.2%) (Figure 2b). Moreover, the spermatozoa from *Gykl1*^−/−^ mice swam with their heads facing backward and twisting tails, a sign of severely impaired spermatozoa motility (Supplementary Movie S1).

Staining of the mitochondrial sheath in the affected spermatozoa revealed obvious abnormalities in the mitochondria. A gap was apparent between the midpiece and tail in the spermatozoa from heterozygous *Gykl1*-KO mice (Figure 2c). An even larger gap was observed in *Gykl1* deficient spermatozoa, with intense mitochondria staining (with the MitoTracker dye) at the end of the mitochondrial sheath. The spermatozoa tail was easily bent at these sites (Figure 2c). The length of the mitochondrial sheath was significantly shorter in the spermatozoa from *Gykl1*- or *Gk2*-deficient mice (For example, 15.25±2.89 μm for Gykl1^−/−^ vs 21.28±1.41 μm for WT) (Figure 2d and e). Additionally, intracytoplasmic spermatozoa injection (ICSI) of *Gykl1*- or *Gk2*-KO spermatozoa led to normal offspring (Supplementary Table S1), suggesting that their infertility may be accounted to defects in fertilization. These results suggest that reduced spermatozoa motility induced by the disrupted mitochondrial sheath is the likely culprit for the infertility of *Gykl1*- *or Gk2*-deficient mice.

### Abnormal mitochondrial sheath and sperm tail fragility are found in spermatozoa from *Gykl1-* and *Gk2-*deficient mice

**Figure 3:**
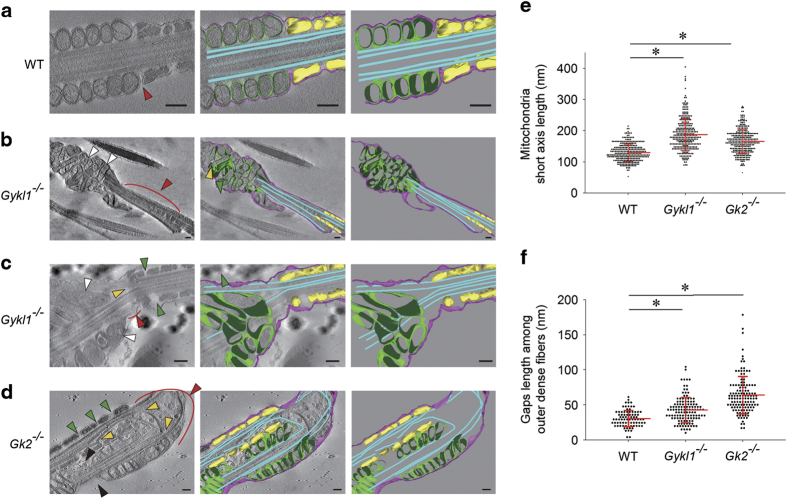
Ultra-structural analysis of the spermatozoa from male *Gykl1* and *Gk2* mutant mice. (**a**–**d**) The spermatozoa were collected from various mice and analyzed by transmission electron microscopy (TEM) coupled with Electron Tomography, which enabled detailed 3D imaging of the midpiece and tail regions. For each group, the middle slice of the electron tomogram is shown on the left, the annotation model on the right, and superimposed tomogram with the annotation model in the middle. Purple indicates the outer cell membrane of the spermatozoa, green the outer membrane of the mitochondria, cyan the doublet microtubule or central microtubule, and yellow the outer dense fibers. White arrows, swollen and misarranged mitochondria. Black arrows, midpiece that lacks mitochondria. Green arrows, gaps between outer dense fibers. Red arrows, gaps between the mitochondrial sheath and outer dense fibers. Yellow arrows, branched or fragile axoneme. Scale bar, 200 nm. (**e** and **f**) The length of the mitochondria short axis (**e**) and the gaps among outer dense fibers (**f**) in the spermatozoa from wild-type and mutant mice were measured and plotted. Over 300 spermatozoa were counted for each group. Error bars represent s.d. Significance was determined by ANOVA. *P<0.05.

Given the possible defects in the mitochondria sheath in the spermatozoa from *Gykl1/Gk2* KO mice, we decided to examine these spermatozoa more carefully using transmission electron microscopy (TEM) coupled with Electron Tomography, which enables more detailed and 3D imaging. As expected, in wild-type (WT) spermatozoa, the mitochondria were neatly lined up along the central axoneme (Figure 3a). However, arrangement of the mitochondria was considerably disordered in *Gykl1*-KO spermatozoa, indicating dysregulated mitochondrial sheath formation (Figure 3b and c, white arrows). Moreover, these mitochondria were of all shapes and sizes. In *Gk2*-KO spermatozoa, the mitochondria were relatively uniform in morphology and size, but they were missing in some areas along the mitochondria sheath, leaving obvious gaps (Figure 3d). The mitochondria in wild-type spermatozoa had a diameter of ~100 nm in their longitudinal section, representing the length of the short axis (Figure 3a and e). In comparison, the mitochondria in *Gykl1*- and *Gk2*-deficient spermatozoa looked large and swollen, nearly doubling the length of the short axis (~180 nm) of wild-type mitochondria (Figure 3e).

The gaps observed under light microscopy (Figure 2a) were clearly visible with TEM (Figure 3a). Compared with wild-type spermatozoa, large gaps between the mitochondrial sheath and outer dense fibers appeared in both *Gykl1* and *Gk2* KO spermatozoa (Figure 3b–d, red arrows). In these regions, the mitochondrial sheath or the outer dense fibers were completely absent, leaving the axoneme 'unprotected'. Moreover, the outer dense fibers had larger gaps in the KO samples (*WT*: 30.51±11.63 nm, *Gykl1*^*−/−*^: 42.77±18.10 nm, *Gk2*^*−/−*^:63.84±26.72 nm), taking on a 'beaded string' look with enlarged gaps within them (Figure 3b–d, green arrows, and Figure 3f). Sperm tail integrity was greatly compromised in the spermatozoa of *Gykl1*- and *Gk2*-deficient mice, with evidence of breaking (Figure 3b–d, yellow arrows). These defects likely affected the activity of the spermatozoa (Table 1), resulting in reproductive abnormalities (Figure 1d and e). Regarding the observation of decreased GK2 in teratozoospermia [31], the *Gykl1* or Gk2 KO mice did show abnormalities in morphology defected spermatozoa, which again raised the possible link between male infertility and Gyk-like proteins. Our analysis thus far indicates that Gykl1 and Gk2 may regulate mitochondrial arrangement and morphology, and the maintenance of sperm tail integrity in spermatozoa.

### Gykl1 and Gk2 contain specific sequences for mitochondrial targeting

**Figure 4:**
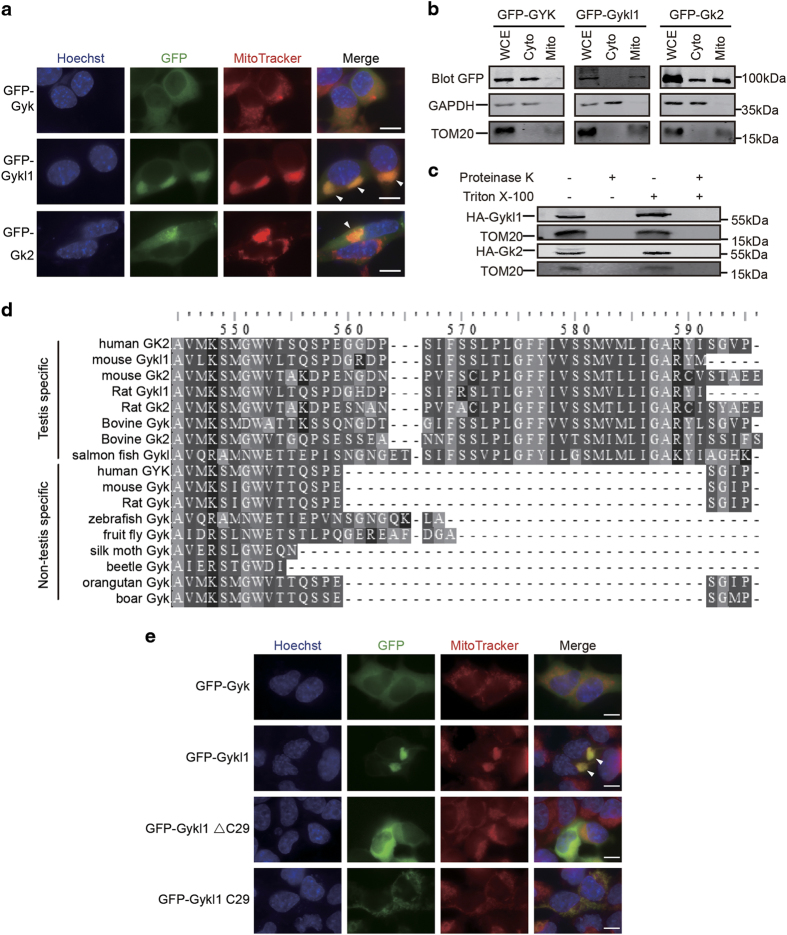
Gykl1 and Gk2 target to the outer membranes of the mitochondria through their unique C-terminal domains. (**a**) HEK293T cells transiently expressing various GFP-tagged proteins were stained with the MitoTracker dye to visualize the mitochondria. Arrows indicate areas of mitochondrial clustering. Hoechst 33342 was used to visualize the nuclei. Scale bar: 10 μm. (**b**) HEK293T cells transiently expressing GFP-tagged proteins were fractionated and blotted with antibodies against GFP. WCE, whole-cell extract. Cyto, cytoplasmic fraction, Mito, mitochondrial fraction. Antibodies against GAPDH and TOM20 served as loading control for the cytosolic and mitochondrial fractions respectively. (**c**) HEK293T cells transiently expressing HA-tagged Gykl1 and Gk2 were used to isolate mitochondrial fractions. The fractions were treated with proteinase K in the presence or absence of Triton X-100 before being immunoblotted with anti-HA antibodies. The antibody against TOM20, a mitochondria outer membrane protein, served as a positive control. (**d**) The C-terminal regions of Gyk-like genes from different species are aligned. Testis-specific genes appear to contain additional sequences in their C-termini compared with Gyk orthologues that are not restricted to testis. (**e**) HEK293T cells transiently expressing GFP-tagged Gyk as well as full-length and truncation mutants of Gykl1 were stained with the MitoTracker dye to visualize the mitochondria, and examined under a fluorescent microscope. Hoechst 33342 was used to visualize the nuclei. Arrows indicate areas of mitochondrial clustering. Scale bar, 10 μm.

Our anti-Gykl1/Gk2 antibody recognizes both Gykl1 and Gk2 (Figure 1; Supplementary Figure S1), and therefore cannot reveal any possible differences in mitochondrial localization between the two proteins. To better understand the localization and function of Gykl1 vs Gk2, we GFP-tagged Gyk, Gykl1 and Gk2 at the N terminus and transiently expressed these proteins in HEK293T cells. As expected, GFP-Gyk exhibited primarily diffused cytoplasmic staining with no apparent mitochondrial localization, which was further confirmed by fractionation experiments (Figure 4a and b). In support of our data with endogenous Gykl1 and Gk2 (Figure 1), GFP-tagged Gykl1 and Gk2 signals clearly overlapped with signals from the MitoTracker dye (Figure 4a), and could be found in mitochondrial fractions (Figure 4b). Treatment with proteinase K led to diminished Gykl1 and Gk2 signals in the mitochondrial fraction (Figure 4c), indicating that these proteins were localized to the mitochondrial outer membrane. It is interesting to note that ectopic expression of Gykl1 and Gk2 led to dramatic clustering of the mitochondria (Figure 4a, white arrows), which was also observed when Gykl1 was overexpressed in HeLa and NIH3T3 cells (Supplementary Figure S3A and B). In comparison, GFP-Gyk remained in the cytoplasm where no mitochondrial rearrangements could be seen.

Of the 17 Gyk family members and orthologues found in the NCBI, several have been shown to be testis-specific by GEO database (Figure 4d). When aligned against one another, all the testis-specific Gyk members and orthologues (including Gykl1 and Gk2) contain an extra C-terminal tail that is absent in the non-testis-specific Gyk orthologues. To test whether this C-terminal region is important for mitochondrial targeting, we generated GFP-tagged truncation mutants of Gykl1 and determined their localization. As shown in Figure 4e, deletion of the last 29 amino acids of Gykl1 (Gykl1ΔC29) abolished its mitochondrial localization, whereas these 29 amino acids alone (Gykl1 C29) were sufficient to target to the mitochondria. Interestingly, no mitochondrial clustering was observed with overexpression of either mutant, indicating that the C-terminus of Gykl1 contains mitochondria-targeting sequences, and the entire Gykl1 sequence is needed for inducing mitochondrial clustering.

### Gykl1 and Gk2 interact with Pld6 to induce PA production and mitochondrial clustering

Gyk phosphorylates glycerol to generate glycerol 3-phosphate, a precursor of PA, the latter of which has been closely linked to mitochondrial clustering [4, 14]. Gyk, Gykl1, and Gk2 share high homology in protein sequences, but do differ in several amino acids, including the Asp17 catalytic site (Supplementary Figure S1A). Previous work found no glycerol kinase activities *in vitro* for either Gykl1 or Gk2 [30]. To determine whether glycerol kinase activities were required for Gykl1/Gk2-induced mitochondrial clustering, we treated cells with the glycerol kinase inhibitor α-thioglycerol before transiently expressing GFP-Gykl1. Even at very high concentrations, α-thioglycerol did not affect mitochondrial clustering (Supplementary Figure S4A and B). Moreover, targeting Gyk to the mitochondria, by fusing the C-terminal mitochondrial targeting sequence of Gykl1 to Gyk, failed to induce mitochondrial clustering (Supplementary Figure S4C). These results suggest that Gykl1-induced mitochondrial clustering does not require the glycerol kinase activity, and that Gykl1 and Gk2 likely work as adapter proteins in regulating mitochondrial dynamics.

**Figure 5:**
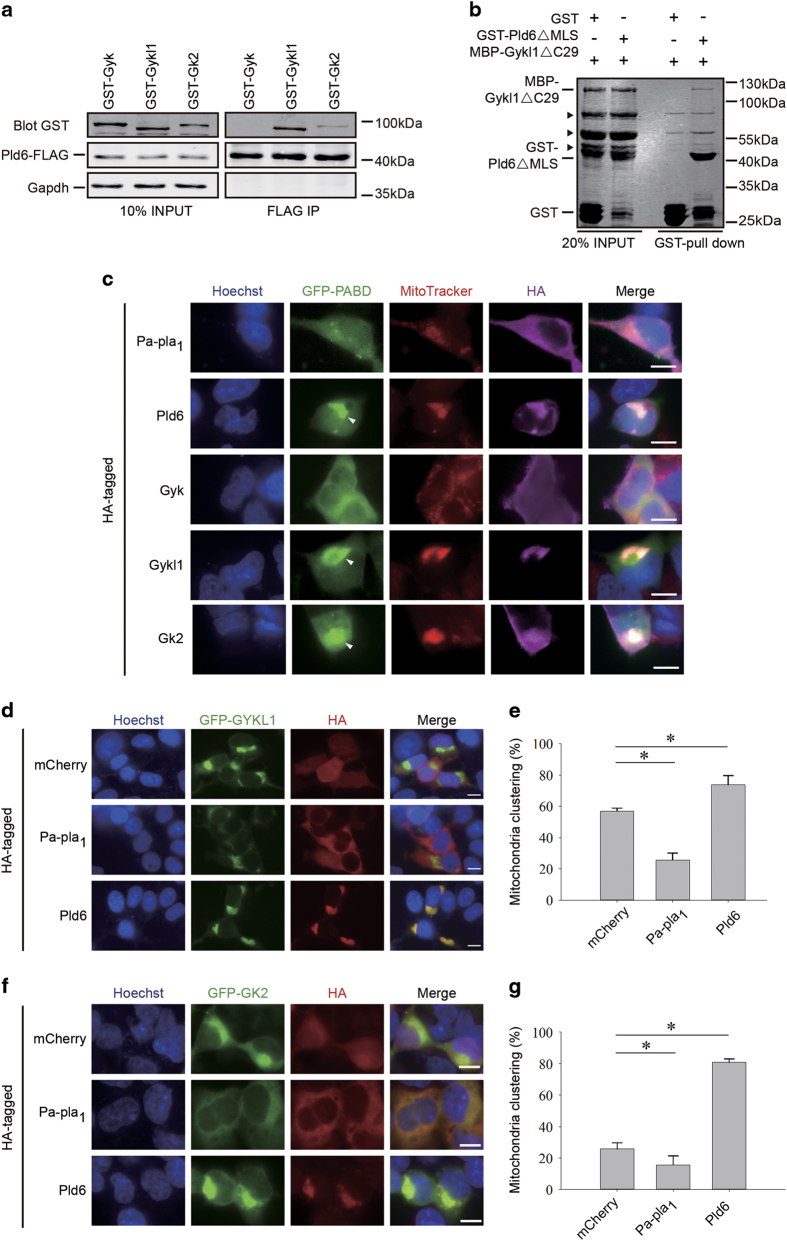
Gykl1/Gk2 interacts with Pld6 and stimulate mitochondrial clustering. (**a**) HEK293T cells transiently co-expressing FLAG-tagged Pld6 with GST-tagged Gyk, Gykl1 or Gk2 were immunoprecipitated (IP) with anti-FLAG antibodies. The immunoprecipitates were blotted with antibodies against GST or FLAG. An antibody against GAPDH was used for loading control. (**b**) Bacterially-purified GST-tagged recombinant Pld6 lacking the mitochondria localization sequence (GST-Pld6ΔMLS), was mixed with purified MBP-tagged recombinant Gykl1 lacking the C-terminal 29 amino acids (MBP-Gykl1ΔC29) for *in vitro* GST pull-down assays. The reaction mixtures were resolved by SDS-PAGE and stained with Coomassie blue. Recombinant GST alone served as a negative control. (**c**) HEK293T cells transiently co-expressing GFP-PABD with HA-tagged Pa-pla1, Pld6, Gyk, Gykl1 or Gk2 were stained with an anti-HA antibody and the MitoTracker dye. Hoechst 33342 was used to visualize the nuclei. PABD is the Raf1 Phosphatidic Acid Binding Domain. Arrows indicate areas of mitochondrial clustering. Scale bar, 10 μm. (**d**) HEK293T cells transiently co-expressing GFP-Gykl1 with HA-tagged mCherry, Pld6 or Pa-pla_1_ were immunostained with an anti-HA antibody. Hoechst 33342 was used to visualize the nuclei. Scale bar, 10 μm. (**e**) Cells from **d** were scored for cells with obvious mitochondrial clustering in three independent experiments (*n*=3) and the percentages of cells with mitochondrial clustering are plotted here. At least 200 cells were counted for each group. Error bars represent s.d. Statistical significance was determined by ANOVA; *P<0.05. (**f** and **g**) HEK293T cells co-expressing GFP-Gk2 with HA-tagged mCherry, Pld6, or Pa-pla_1_ were similarly examined (**f**) and analyzed (**g**) as described in **d** and **e**. At least three independent experiments were performed with >200 cells counted in each group. *P<0.05. Scale bar, 10 μm.

To further probe the mechanisms of Gykl1/Gk2-mediated mitochondria regulation, we assessed possible interactions between Gykl1/Gk2 and several proteins known to regulate mitochondrial dynamics. MFN1 and DRP1 can directly control mitochondrial fusion and fission [14]. LIPIN1 was reported to degrade PA and generate DAG during the process of mitochondria fission [16], and Pa-pla_1_ can induce mitochondrial fragmentation by turning PA into LPA [1]. Pld6 (MitoPLD) has key roles in generating PA from CL and mitochondria clustering [4]. GST pull-down experiments found no interactions between Gyk and any of the regulators examined (Supplementary Figure S5A). Neither could we detect interactions of Gykl1/Gk2 with Mfn1, Drp1, Lipin1 or Pa-pla_1_ (Supplementary Figure S5B and C). However, both Gykl1 and Gk2, but not Gyk, were able to co-precipitate with Pld6 (MitoPLD) (Figure 5a; Supplementary Figure S5). This interaction appeared to be independent of mitochondrial localization because deleting the mitochondrial localization sequence (MLS) from both Gykl1 and Pld6 (Gykl1ΔC29 and Pld6ΔMLS) did not affect their interaction *in vitro* (Figure 5b). Taken together, these results raise the possibility that the Gyk-like proteins Gykl1/Gk2 may regulate mitochondrial dynamics through Pld6 and the PA biogenesis pathway.

When Pld6 is overexpressed in cells, mitochondrial PA synthesis is upregulated, which leads to increased mitochondrial clustering [4]; Pa-pla_1_, on the other hand, can reduce mitochondrial PA levels, resulting in mitochondrial fragmentation [1]. To determine whether Gykl1/Gk2-induced mitochondrial clustering involves the PA synthesis pathway, we ectopically expressed various proteins in HEK293T cells and monitored intracellular PA levels using GFP-tagged PA-binding domain of Raf1 (GFP-PABD) [40]. As expected, overexpression of Pld6 led to clustered signals of GFP-PABD that also co-stained with MitoTracker (Figure 5c). In contrast, expression of Pa-pla_1_ did not alter the distribution of GFP-PABD signals. Similar to Pld6-expressing cells, cells expressing Gykl1 and Gk2, but not Gyk, also displayed elevated GFP-PABD signals and clustering in the mitochondria (Figure 5c). If this Gykl1/Gk2-induced mitochondrial clustering involved PA synthesis, then changing mitochondrial PA levels (e.g., through Pld6/Pa-pla_1_ expression) should also impact mitochondrial dynamics in Gykl1/Gk2-expressing cells. Indeed, addition of Pld6 or Pa-pla_1_, led to increased or decreased percentages of Gykl1/Gk2-expressing cells showing mitochondrial clustering, respectively (Figure 5d–g). These results suggest that Gykl1/Gk2 interacts with Pld6, trigger PA synthesis, and induce mitochondrial clustering in cells.

### Gyk-like proteins specifically induce mitochondrial clustering via Pld6

**Figure 6:**
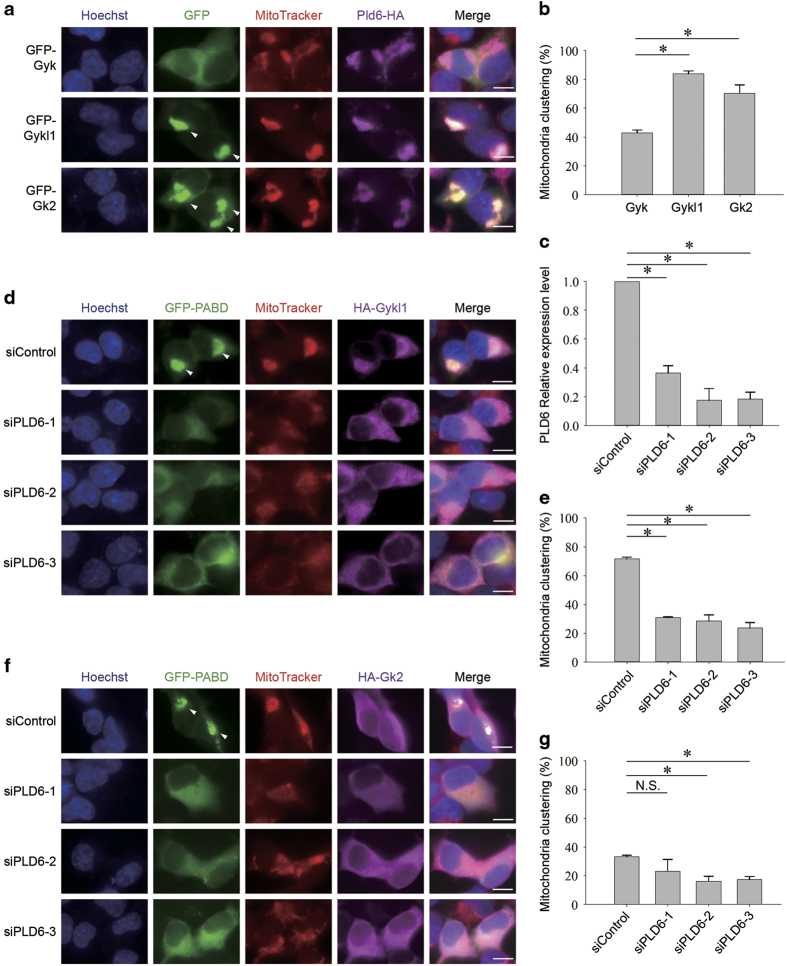
Gykl1/Gk2-mediated mitochondrial clustering is PA dependent. (**a**) HEK293T cells transiently co-expressing HA-tagged Pld6 with GFP-tagged Gyk, Gykl1 or Gk2 were stained with an anti-HA antibody and the MitoTracker dye. Hoechst 33342 marks the nuclei. Arrows indicate areas of mitochondrial clustering. Scale bar, 10 μm. (**b**) Cells from **a** were scored for mitochondrial clustering and plotted for the percentage of cells with mitochondrial clustering. Three independent experiments were carried out with >200 cells per group. Error bars represent s.d. Statistical significance was determined by ANOVA; *P<0.05. (**c**) HEK293T cells were transfected with three different siRNA oligos against Pld6. At 45 h post transfection, the cells were harvested for RT-qPCR analysis. A scramble siRNA was used as a negative control. The expression level of Pld6 was calculated for each group and plotted. Error bars represent s.d. (*n*=3). Statistical significance was determined by ANOVA; *P<0.05. (**d**) HEK293T cells co-expressing GFP-PABD and HA-Gykl1 were transfected with the Pld6 siRNAs from **c**. At 45 h post transfection, the cells were harvested for staining using antibodies against HA and the MitoTracker dye. Hoechst 33342 marks the nuclei. Arrows indicate areas of mitochondrial clustering. Scale bar, 10 μm. (**e**) Cells from **d** were scored and plotted as described in **b**. Three independent experiments were carried out with >200 cells per group. Error bars represent s.d. Statistical significance was determined by ANOVA; *P<0.05. (**f** and **g**) HEK293T cells co-expressing GFP-PABD and HA-Gk2 were similarly transfected with Pld6 siRNAs, and examined (**f**) and analyzed (**g**) as described in **d** and **e**. Three independent experiments were carried out with >200 cells per group. Error bars represent s.d. Statistical significance was determined by ANOVA. *P<0.05; Scale bar: 10 μm.

Next we hypothesized if Gykl1/Gk2 could further stimulate PA synthesis and induce mitochondrial clustering when Pld6 is overexpressed. As expected, when we reciprocally co-expressed Gykl1 or Gk2 in cells ecto-expressing Pld6, induced mitochondrial clustering was clearly visible (~70–80%), compared with cells co-expressing Gyk (~40%) (Figure 6a and b). Our results thus far support the notion that Gykl1 and Gk2 can induce mitochondrial clustering through interactions with Pld6 and upregulation of PA synthesis in the mitochondria. We further tested this model by knocking down Pld6 in Gykl1/Gk2-expressing cells. All three siRNAs achieved >60% knockdown efficiency (Figure 6c). Importantly, inhibition of Pld6 in these cells diminished GFP-PABD signals in the mitochondria, indicating lower PA levels, and resulted in more diffused staining of MitoTracker, indicating less mitochondrial clustering (Figure 6d–g). Similar results were also obtained using HeLa cells overexpressing Gykl1 (Supplementary Figure S6A–C). These results reinforce the notion that Gykl1/Gk2-induced mitochondrial clustering and PA synthesis depends on Pld6.

Unlike mouse, human appears to have only one Gyk-like protein—GK2. The human homolog of mouse Gykl1 and Gk2 could also specifically localize to the spermatozoa midpiece (Supplementary Figures S1B and S7A). Furthermore, GK2 could also interact with hPLD6 (Supplementary Figure S7B). As was the case with mouse Gykl1 and Gk2, ectopic expression of GK2 similarly led to changes in mitochondrial dynamics, and this regulation again depended on hPLD6 and involved PA biogenesis in the mitochondria (Supplementary Figure S7C–F), indicating the regulation of PA biosynthesis as central to mitochondrial clustering induced by Gyk-like proteins. Mitochondrial function was also regulated by Gyk-like proteins. ATP concentration in Gykl1 or Gk2-defciency spermatozoa was significantly higher than the wild-type one (Supplementary Figure S8A). Cells overexpressing Gykl1, Gk2 or GK2 had decreased ATP level (Supplementary Figure S8B). Regarding that increased GK2 may correlate to asthenozoospermia [32], we suspected that increased GK2 could induce abnormal mitochondrial function or ATP deficiency then contribute to asthenozoospermia. Our studies here provide the first evidence for the evolutionarily conserved functions of Gyk-like proteins, which activate Pld6 to induce mitochondrial PA biogenesis and clustering, processes that are crucial for mitochondrial ATP production, spermiogenesis and male fertility.

## Discussion

Spermatogenesis is essential to sexual reproduction and is highly regulated. Of the numerous genes known to be involved in the process of spermatozoa production, several have testis-specific isoforms with important functions in germ cells [41,42,43,44]. Unlike *Gyk*, the *Gyk*-like intronless retroposons *Gykl1* and *Gk2* are expressed primarily in testis [30]. Here, we show for the first time that Gykl1 and Gk2 are in fact localized to the mitochondria in round and elongating spermatids and retained within the mitochondrial sheath in spermatozoa. While *Gyk-*deficient mice die within 3–4 days after birth, exhibiting postnatal growth retardation, autonomous glucocorticoid synthesis, and abnormal fat metabolism [25], *Gykl1*^*−/−*^ and *Gk2*^*−/−*^ mice were viable. However, these mutant mice display specific reproductive defects. For example, *Gykl1*^+/−^ males showed subfertility and *Gykl1*^−/−^ males were infertile, and their spermatozoa exhibiting unregulated ATP level and reduced motility. Interestingly, *Gykl1*^*+/−*^ male mice were subfertile, while *Gk2*^*+/−*^ male mice display completely normal fertility. *Gk2* KO mice showed similar reproductive defects, indicating functional similarities between Gk2 and Gykl1. Still, it needs more in depth investigations to define any function similarity between Gykl1 and Gk2.

**Figure 7:**
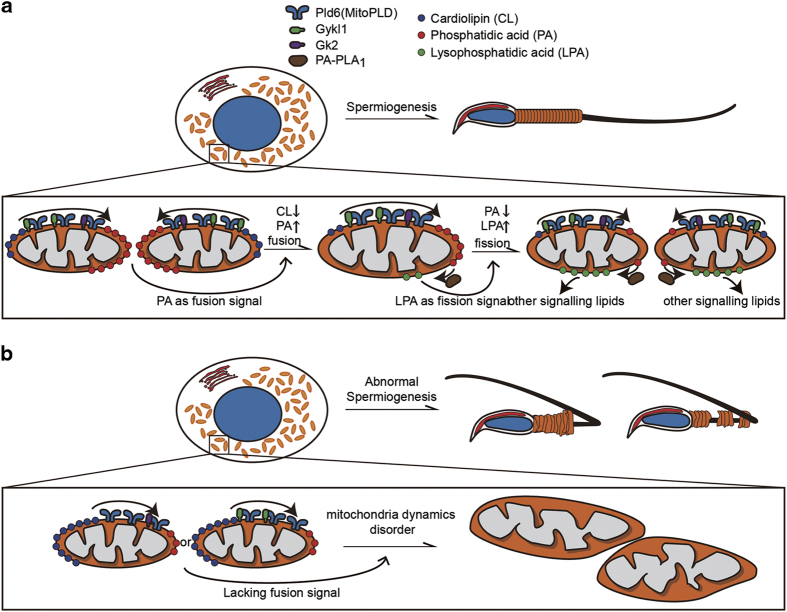
A model for the regulation of mitochondrial dynamics in spermiogenesis. (**a**) During normal spermiogenesis, Pld6 (MitoPLD) generates PA from CL. Gykl1 is expressed in round spermatids. Gykl1 and Gk2 physically interact with Pld6 and facilitate Pld6-mediated PA biogenesis, which further induces mitochondrial fusion. Mitochondrial PA is then converted into LysoPA by Pa-pla_1_, which induces mitochondrial fission. PA, LysoPA and other signaling lipids together regulate mitochondrial dynamics and contribute to mitochondrial sheath formation during spermiogenesis. (**b**) In spermatids lacking Gykl1 or Gk2, PA, LPA and other lipids that regulate mitochondria dynamics are reduced, which contributes to disordered mitochondrial morphology, abnormal regulation of mitochondrial dynamics and disrupted midpiece formation.

Mitochondrial sheath abnormalities in the spermatozoa have been observed in a number of mutant mice, including those deficient for *SEPP1, Sept4, KLC3, SLIRP, GPx4, Pa-pla*_*1*_ or *Guf1* [1,7,45,46,47,48,49]. How SEPP1, Sept4, SLIRP and Guf1 maintain mitochondrial sheath integrity remain unclear [45, 46, 48, 49]. KLC3 has been proposed to regulate mitochondrial trafficking in spermatids [7], and Pa-pla_1_ is important in phospholipid metabolism during spermatogenesis [1]. In this study, we have provided evidence that links Gykl1 and Gk2 to mitochondrial clustering and PA generation, and supports the notion that Gyk-like proteins may regulate mitochondrial dynamics through facilitating PA biogenesis (Figure 7) in spermatogenesis. The absence of Gykl1 or Gk2 greatly altered mitochondrial morphology and dynamics during spermiogenesis, which negatively impacted sperm tail structure and sperm activity (Figure 7). Mechanistically, Gykl1 and Gk2 appear to cooperate with and depend on Pld6 for their function. It will be interesting to determine whether dysfunction of Gykl1 and/or Gk2 disrupts the interactions between Gykl1/Gk2 and Pld6 and contributes to the spermatozoa defects.

Mitochondrial dynamics is intimately linked to PA synthesis, which occurs through the hydrolysis of CL, phosphorylation of DG, or acylation of LPA. LPA is generated from acylation of glycerol 3-phosphate, a product of Gyk-mediated glycerol phosphorylation [1, 34,50,51,52]. The exact roles that PA and LPA have in the pathways by which Gyk-like proteins modulate mitochondrial dynamics remain to be uncovered. In this study, we found that inhibiting glycerol kinase activities in cells had no effects on Gykl1-mediated mitochondrial clustering. This result is supported by the findings of an absence of male reproductive defects in mice deficient in GPAT4, the testis-specific glycerol 3-phosphate acyltransferase that converts glycerol 3-phosphate into LPA [53,54,55]. These data combined suggest that glycerol kinase activities are not required for the regulation of mitochondrial dynamics by Gyk-like proteins.

Testis-specific *Gyk*-like genes have been identified in multiple species, although humans have only one (GK2) vs two for mice and rats. We have found evolutionary conservation in both sequence and function for human and mouse homologs, such as the interaction with Pld6 and the regulation of mitochondrial dynamics. While mouse Gykl1 and Gk2 appear to carry out similar function, they are both indispensable since knocking out either gene affected male fertility. How these two proteins with high sequence identity, overlapping localization and shared functionality complement each other during spermatogenesis warrants further investigation. Such studies will also have important implications for humans because GK2 likely assumes the roles of both mGYKL1 and mGK2. Whether this is the case or other as yet unidentified factors that functionally complement GK2 do exist should be examined in more detail. Notably, dysregulated GK2 expression has been linked to teratozoospermia and asthenozoospermia [31, 32]. Defects in mitochondrial sheath and damages in sperm tail in *Gykl1* or *Gk2* KO mice mimic the case in GK2-related teratozoospermia. Also, the unregulated ATP level we observed indicates a potential mechanism of GK2-related asthenozoospermia that could be partially caused by defects in mitochondrial ATP production. Our findings here point to GK2 as an important player in male fertility. It still needs further investigation on whether GK2 has unexpected enzymatic activities. In depth study on the role of GK2 in spermatogenesis may provide new insights into male fertility.

## Materials and methods

### Plasmid construction

The cDNAs of full-length *Mfn1, Drp1, Lipin1, Pa-pla1, Pld6, Gyk, Gykl1, Gk2* and *GK2* were cloned into the pENTR/D-TOPO via the NotI and AscI restriction sites. Various truncation mutants are *Gykl1*Δ*C(1–1560), Gykl1C29(1561–1607)* and *Pld6*Δ*MLS(115–663)*. The cDNAs were Gateway cloned into various mammalian expression vectors containing Flag-HA tag (pLentiFHA or pMSCVFHA), SFB tag (S-tag, streptavidin-binding protein, Flag) (pLentiSFB or pBabeSFB), GST tag (pDEST27), or GFP tag (pHAGE-GFP). *Raf1-PABD(1165–1269)* was cloned and ligated to IRES with *Pa-pla1, Pld6, Gyk, Gykl1 and GK2.*

### Generation of genetically-modified mice

Mice were housed under climate-controlled (22±1 °C) conditions in a designated pathogen-free animal facility with a 14-h-light/10-h-dark cycle at Sun Yat-Sen University. The Institutional Animal Care and Use Committee of Sun Yat-Sen University, P.R. China have approved all the experimental protocols concerning the handling and husbandry of the mice.

### Generation of *Gykl1* and *Gk2* knockout mice by CRISPR/Cas9

The pT7-3XFlag-hCas9 plasmid was linearized with PmeI for the synthesis of Cas9 mRNA using the mMACHINE T7 ULTRA kit (Life Technologies, Waltham, MA, USA). The pDR274 vector encoding the desired gRNA sequences (*Gykl1* gRNA: AGCGGGAAACTACGATAGTC; *Gk2* gRNA: TTGGAAGGCGTGCCAATATC) was *in vitro* transcribed using the MEGAshortscript T7 kit (Life Technologies). The Cas9 mRNA and the gRNAs were subsequently purified with the MEGAclear kit (Life Technologies), resuspended in RNase-free water, and quantified using NanoDrop-1000. The Cas9 mRNA and gRNA mixture was then injected into 0.5-day zygotes of C57BL/6J mice. Three hours after injection, the injected zygotes were transplanted into the oviduct of 0.5-day pseudopregnant mothers. CRISPR/Cas9 knockout mice were bred with wild-type mice for more than three generations to minimize off-target effects. Mouse genotyping was done by PCR and sequencing of tail-snips using the Mouse Genotyping Kit (KAPA biosystems, KK7302) following the manufacturer’s instruction.

### Immunoblotting

For tissues samples, tissues were homogenized in ice-cold RIPA buffer (50 mM Tris-HCl pH 8.0, 150 mM NaCl, 1% NP-40, 1 mM EDTA pH 8.0, 0.5% sodium deoxycholate, 0.1% SDS) supplemented with protease inhibitor cocktail (Sigma, St Louis, MO, USA, P8340). The homogenates were clarified by centrifugation and quantified with BCA Protein Assay Kit (Pierce, Waltham, MA, USA, 23225). For cultured cells and protein, 1× SDS-PAGE loading buffer was added and heated at 95 °C for 20 min. Sample were then subjected to SDS-PAGE and transferred to membrane. After blocking by 5% non-fat milk, proteins were detected with antibodies. Rabbit anti-Gykl1/Gk2 antibody (1:10 000), rabbit anti-GST antibody (1:10 000), mouse anti-GAPDH antibody (1:5 000), mouse anti-TOM20 antibody (1:5 000), rabbit anti-HA antibody (1:5 000), mouse anti-FLAG antibody (1:5 000), rabbit anti-DYKDDDDK antibody (1:5 000), goat anti-mouse IgG (1:10 000), goat anti-rabbit IgG (1:10 000). The membrane was scanned using the Licor Odyssey system.

### Histological analysis

For hematoxylin and eosin staining, Bouin’s solution-fixed and paraffin-embedded testis sections (4 μm) were sequential rehydrated and stained with hematoxylin and eosin.

### Immunofluorescent microscopy

For testis sections, testis sections were rehydrated and microwaved for 10 min in citric buffer (10 mM citric acid monohydrate, 10 mM sodium citrate). For spermatozoa, spermatozoa was diluted in H_2_O containing 150 nM Mitotracker Red CMXRos at 25 °C for 15 min. The spermatozoa was speared and dried on a slide, then fixed with 4% formaldehyde in PBS at 25 °C for 30 min. For adhered cells, samples were fixed in 4% formaldehyde in PBS at 25 °C for 30 min. Permeabilize by 0.5% Trition-X, 20 mM HEPES, 50 mM NaCl, 3 mM MgCl_2_, 300 mM. Then samples were incubated with primary antibodies in PBS (pH 7.4) containing 5% BSA for 1 h at room temperature. After three times washes by PBS, the samples were incubated with secondary antibodies in PBS (pH 7.4) containing 5% BSA and 10 μgml^−1^ Hoechst 33342 (Invitrogen, H3570) for 1 h at room temperature. Antibodies and dilutions: rabbit anti-Gykl1/Gk2 antibody (1:1 000), mouse anti-SYCP3 (1:200), goat anti-PLZF antibody (1:500), mouse anti-HA antibody (1:1 000), Alexa Fluor 488-conjugated donkey anti-mouse IgG (1:2 000), Alexa Fluor 488-conjugated donkey anti-rabbit IgG (1:2 000), Alexa Fluor 555-conjugated donkey anti-mouse IgG (1:2 000), Alexa Fluor 555-conjugated donkey anti-rabbit IgG (1:2 000), Alex Fluor 647-conjugated donkey anti-mouse IgG (1:1 000) and Alexa Fluor 647-conjugated donkey anti-goat IgG (1:1 000, Life Technologies).

### Breeding analysis

Indicated numbers of 5 month old WT and KO male mice were subjected for breeding analysis. Each male mouse mated with a WT female (8 weeks) mouse until observation of vaginal plug. Litter number was counted 14–24 days after vaginal plug formation. Any offspring producing was marked as successful pregnancy.

### Epididymal sperm analysis

The cauda epididymis was obtained from 19-week-old mice. Sperm from the cauda epididymis was collected into 3% BSA in DMEM/F-12 (Gibco, Waltham, MA, USA, 11039-021) at 37 °C. Sperm motility was monitored using a computer-assisted sperm analysis system (Sperm Class Analyzer, MICROPTIC S.L.).

### Electron tomography and image processing

Electron tomography of the semi-ultrathin sections was conducted using Tomography in the FEI Tecnai F20 microscope (FEI, Hillsboro, OR, USA) operated at 200 kV. The data were recorded with a 4 K×4 K CCD camera (Eagle, FEI, USA), and the tilt angles ranged from −60° to +60° with a 2° interval. The nominal magnifications of the tomograms were 7 800×, 11 500× and 14 500×, and the corresponding final Å/pixel values were 15.4, 19.6 and 28.3, respectively. The IMOD package (Kremer *et al.*, 1996) was used for reconstruction, segmentation and rendering.

### Intracytoplasmic spermatozoa injection

Six-week-old female mice were superovulated with PMSG and hCG. Unfertilized oocytes were harvested 14 h after injection of hCG. ^−/−^ Sperm heads were then injected into the cytoplasm of the unfertilized oocytes by microinjection. The fertilized oocytes were cultured overnight in KSOM medium. The next day, 2-cell embryos were transplanted into a pseudopregnant mother.

### Mitochondrial fractionation

Mitochondria were prepared as previously described (Wieckowski, *et al.*, 2009). Briefly, cells were harvested and washed with PBS before addition of ice-cold hypotonic buffer (225 mM mannitol, 75 mM sucrose, 0.05 mM EDTA, 30 mM Tris-HCl, pH 7.4). The cells were then homogenized by 75 strokes of a Telflon homogenizer. Unbroken cells and nuclei were removed by centrifugation at 600 g for 5 min at 4 °C. Mitochondrial and cytosolic fractions were separated by centrifugation at 9 000 *g* for 10 min at 4 °C. The resulting mitochondria were treated in the presence or absence of 0.5% Triton X-100 or 100 μg protease K on ice for 30 min. The reaction was stopped by adding 5× SDS-PAGE loading buffer for further analysis.

### RT-PCR and quantitative RT-PCR

Total RNA from various mouse tissues were extracted with TRIzol reagent (Invitrogen, Waltham, MA, USA, 15596-018). First-strand cDNAs were synthesized using RevertAid First Strand cDNA Synthesis Kit (Thermo Scientific, Waltham, MA, USA, #K1621). RT-PCR was performed using TAKARA Taq (TAKARA, Kusatsu, Shiga, Japan, R001A). Reaction mixture was heated at 95 °C for 3 min, followed by 25 cycles of denaturing at 95 °C for 20 s, annealing at 60 °C for 30 s and extension at 72 °C for 45 s with a final extension at 72 °C for 5 min.

RT-PCR primers: Gyk RT FP: CTACAAGGGCAGGTTGAGTT, Gyk RT RP: GATGGTCTGTGCATGGTGGC, Gykl1 RT FP: TGCGGGCTCACCCAGTT, Gykl1 RT RP: CGACCTTCACTTTCCTCGACAT, Gk2 RT FP: AAATGTTTGGAGTCTTGAACCTGAA, Gk2 RT RP: GTATTTGTAGGGGCCTCATGGGTTC, mouse β-actin RT FP: GTCCCTCACCCTCCCAAAAG, mouse β-actin RT RP: GCTGCCTCAACACCTCAACCC.

Quantitative PCR was performed using GoTaq qPCR Master Mix (Promega, Madison, WI, USA, A6002). Reaction mixture was heated at 95 °C for 10 min. 40 cycles amplification of 95 °C for 15 s and 60 °C for 60 s. Data were analyzed by The StepOnePlus™ System (ABI).

Quantitative PCR primers: Gykl1 q FP: TGCGGGCTCACCCAGTT; Gykl1 q RP: CGACCTTCACTTTCCTCGACAT mGapdh q FP: CATGAGAAGTATGACAACAGCCT, mGapdh q RP: AGTCCTTCCACGATACCAAAGT, hPLD6 q FP: TCGTCACCGACTGCGACTA, hPLD6 q RP: CACGATGGCAAACTTGTGATG hGAPDH-q-FP: CATGAGAAGTATGACAACAGCCT, hGAPDH-q-RP: AGTCCTTCCACGATACCAAAGT.

### Cell culture, transfection and coimmunoprecipitation

HEK293T, HeLa and NIH3T3 cells were cultured in DMEM with 4.5 g l^−1^ glucose, l-glutamine and sodium pyruvate (CORNING, 10-013-CVR) with 10% fetal bovine serum under standard condition. Plasmids were transfected using PEI (P3143, Sigma) or Lipofectamine 2000 (Invitrogen, 11668-019) according to manufacturer’s instruction. Cells were collected and lysed in ice-cold IP buffer (50 mM Tris-HCl, pH 8.0, 100 mM NaCl, 1 mM EDTA, 0.5% NP-40, 10% Glycerol) supplemented with protease inhibitor cocktail at 4 °C for 30 min. Cell lysis was clarified and incubated with M2-affinity gel (A2220-5ML) at 4 °C for 60 min. The gel was washed and eluted by 1× SDS-PAGE loading buffer for immunoblotting analysis.

### RNA interference

The siRNA targeting sequences used in HeLa or HEK293T cells: siControl: scramble, siPLD6-1: GCTACATGCATCACAAGTT, siPLD6-2: CGCAAGCCATCCAGAACAA, siPLD6-3: CAGCGAAAGCCAAACCTAA (RIBOBIO). Final concentration of 50 nM of siRNA was used to transfect HeLa or HEK293T cells in a reverse transfection manner according to manufacturer’s instruction. Cells were collected or fixed 45 h post-transfection for further analysis.

### Protein purification

mPLD6ΔMLS(115–663) was sub-cloned into a modified pGEX5-2 vector through NotI and AscI restriction sites. The resulting plasmid was then transformed into BL21 (DE3). The GST fusion protein was expressed by adding 0.1 mM IPTG, and the bacteria were further cultured at 16 °C for 16 h until the OD600 reached 1.0. GST-mPLD6ΔMLS was purified with a Glutathione Sepharose 4B column (GE Healthcare, 17-0756-01) in lysis buffer (50 mM Tris-HCl, pH 8.0, 300 mM NaCl) according to the manufacturer’s instructions. Gykl1ΔC29 (1–1 560) was sub-cloned into a modified pDest17 vector containing an MBP tag through NotI and AscI restriction sites. The resulting plasmid was then transformed into BL21 (DE3). The His-MBP fusion protein was expressed by adding 0.1 mM IPTG, and the bacteria were further cultured at 16 °C for 16 h until the OD600 reached 1.0. His-MBP-Gykl1ΔC29 was purified through a Ni Sepharose FF column (GE Healthcare, Pittsburgh, PA, USA, 17-5318-01) in lysis buffer 2 (50 mM Tris-HCl, pH 8.0, 300 mM NaCl, 20 mM imidazole) according to the manufacturer’s instructions. The purified proteins were mixed at 4 °C for 12 h. The mixture was then clarified by centrifugation and incubated with Glutathione Sepharose 4B for 30 min. The resin was washed with lysis buffer (50 mM Tris-HCl, pH 8.0, 300 mM NaCl). Protein was eluted by 1× SDS-PAGE loading buffer for SDS-PAGE and Coomassie staining.

### ATP level determination

The ATP values of cells were determined using Beyotime Enhanced ATP Assay Kit (S0027). The results were normalized to cell numbers of the samples.

### Statistical analysis

The statistical significances between the mean values for different samples were examined using one-way ANOVA with the Holm-Sidak test when the data were normally distributed with equal variance (SigmaPlot 12.5). Otherwise, Kruskal–Wallis ANOVA on ranks was used (SigmaPlot 12.5). Percentage data were arc-sine-transformed before testing. The data were considered significant when *P*<0.05 (*), 0.01 (**) or 0.001 (***).

## Figures and Tables

**Table 1:** Impact of Gykl1 or Gk2 knockout on spermatozoa mitility

*Parameter*	*WT*	*Gykl1*^*+/−*^	*Gykl1*^*−/−*^	*Gk2*^*+/−*^	*Gk2*^*−/−*^
Motile (%)	52.75±8.6	39.82±7.57	26.05±4.84^***^	50.91±12.79	21.12±6.27^***^
CurviLinear velocity (μms^−1^)	200.41±19.79	156.59±9.27^**^	132.19±20.06^**^	174.87±42.55	106.91±16.6^***^
Straight-line velocity (μm^−1^)	62.51±6.66	27.75±8.87^***^	22.67±9.33^***^	50.5±10.97*	24.54±10.51^***^
Average path velocity (μms^−1^)	95.59±7.56	56.48±12.05^***^	46.24±10.94^***^	81.08±19.12	43.69±13.45^***^
Amplitude of lateral head movement (μm)	11.37±1.17	6.62±0.81*	4.53±2.55*	8.37±2.47	4.48±2.67*
Beat-cross frequency (Hz)	6.12±0.41	4.04±1.39*	2.42±1.38^**^	6.71±0.73	1.97±1.95^**^

Abbreviations: GyK, glycerol kinase; WT, wild type.

Parameters of spermatozoa were analyzed by Computer-Assisted Spermatozoa Analysis system.

Data are

mean±s.d., *n*=3–7, *P<0.05, **P<0.01, ***P<0.001.
