# Supplementary material for: Glycerol kinase-like proteins cooperate with Pld6 in regulating sperm mitochondrial sheath formation and male fertility
Source: Cell Discov. 2017 Aug 22;3:17030–. doi: 10.1038/celldisc.2017.30 (PMC5566117; doi:10.1038/celldisc.2017.30)
Supplement: Supplementary Information [file celldisc201730-s1.pdf]

Glycerol Kinase-Like Proteins Cooperate with Pld6 in Regulating Sperm Mitochondrial Sheath Formation and Male Fertility

Yuxi Chen, Puping Liang, Yan Huang, Minyan Li, Xiya Zhang, Chenhui Ding, Zhen Zhang, Xueqing Zhang, Yuanzhu Gao, Qinfeng Zhang, Shanbo Cao, Haiyan Zheng, Dan Liu, Zhou Songyang*, and Junjiu Huang*

Supplementary information includes 8 Figures. (Figs S1-S8) and 1 Table (Table S1).

Supplementary Figure S1. Expression pattern of G*ykl1* and *Gk2.* (**A**) Protein sequence alignment of GYK, Gyk, Gykl1, Gk2 and GK2. (**B**) Expression analysis of *Gyk*, *Gykl1* and *Gk2* mRNA in various tissues from C57BL/6J mice by RT-PCR. (**C**) Immunoblotting of overexpressed C-terminally SFB-tagged GFP, Gykl1 and Gk2 protein in HEK293T cells. Samples were blotted with a FLAG antibody, the Gykl1/Gk2 antibody or a Gapdh antibody (SFB tag: S-tag, streptavidin-binding protein tag and FLAG tag). (**D**) Immunoblotting of Gykl1 and Gk2 protein in testes of C57BL/6J mice at different developmental stages. dpp: day post-partum. (**E**) Immunostaining of Gykl1/Gk2 in testes from 6dpp, 21dpp and 35 dpp C57BL/6J mice. Scale bar, 10 μm. (**F**) Immunostaining of Gykl1/Gk2, SYCP3 and PLZF in testes from 35 dpp C57BL/6J mice. Scale bar: 10 μm.

Supplementary Figure S2. Normal morphology of testes from *Gykl1/Gk2*-deficient mice. (**A**) Testis morphology of wild-type, *Gykl1 and Gk2* mutant testes. Scale bar: 0.5 cm. (**B**) Testis weight to body weight index. Each grey dot represents an individual mouse (mean ± S.D.; n=3-10). (**C**) H&E staining of seminiferous tubules from wild-type, *Gykl1 or Gk2*-deficient mice. Scale bar: 10 μm.

Supplementary Figure S3. Gykl1/Gk2 induced mitochondrial clustering in HeLa and NIH3T3 cells. (**A**) MitoTracker staining of HeLa cells transfected with GFP-Gyk or GFP-Gykl1. (**B**) MitoTracker staining of NIH3T3 cells transfected with GFP-Gyk or GFP-Gykl1. Scale bar: 10 μm.

Supplementary Figure S4. Glycerol kinase activity did not affect Gykl1-induced mitochondrial clustering. (**A**) MitoTracker staining of HEK293T cells transfected with GFP-Gykl1 and treated with various concentrations of 1-thioglycerol. (**B**) Statistical analysis of mitochondrial clustering severity for HEK293T cells transfected with GFP-Gykl1 and treated with various concentrations of 1-thioglycerol (mean ± S.D.; n=2 independent experiments; cells counts>100). Scale bar: 10 μm. (**C**) Subcellular localization analysis for the C-terminal 29 amino acids of Gykl1 and Gyk-Gykl1-C29 fusion. Scale bar: 10 μm.

Supplementary Figure S5. Interaction between GYK family proteins with mitochondrial dynamics-related proteins. (**A**) GST-pull down of GST-tagged GYK with FLAG-tagged GFP, Mfn1, Drp1, Lipin1, Pld6 and Pa-pla$_1$ in HEK293T cells. (**B**) GST-pull down of GST-tagged Gykl1 with FLAG-tagged GFP, Mfn1, Drp1, Lipin1, Pld6 and Pa-pla$_1$ in HEK293T cells. (**C**) GST-pull down of GST-tagged Gk2 with FLAG-tagged GFP, Mfn1, Drp1, Lipin1, Pld6 and Pa-pla$_1$in HEK293T cells.

Supplementary Figure S6. Gykl1 induces mitochondrial clustering through PLD6 in HeLa cells. (**A**) Statistical analysis of PLD6 knock down efficiency in HeLa cells (mean ± S.D.; n=3 independent experiments; *P<0.05). (**B**) Immunostaining of PLD6 knock down HeLa cells expressing GFP-Gykl1. (**C**) Statistical analysis of mitochondrial clustering for PLD6 knock down HeLa

cells expressing GFP-Gykl1 (mean ± S.D.; n=3 independent experiments; cells counts>100; *P<0.05). Scale bar: 10 μm.

Supplementary Figure S7. Mitochondrial localization and PLD6-dependent mitochondrial clustering of GK2. (**A**) Immunostaining of GK2 in human spermatozoa. (**B**) Streptavidin pull down of HA-tagged hPLD6 with GST-tagged GYK or GK2 in HEK293T cells. (**C**) Immunostaining of HEK293T cells coexpressing GFP-GK2 with mCherry, PLD6 or PA-PLA_1. (**D**) Statistical analysis of mitochondrial clustering severity for GFP-GK2-expressing HEK293T cells transfected with mCherry, PLD6 or PA-PLA_1 (mean ± S.D.; n=3; cells counts>200; *P<0.05). (**E**) Immunostaining of PLD6 knockdown HEK293T cells coexpressing GFP-GAPD and HA-tagged GK2. (**F**) Statistical analysis of mitochondrial clustering in PLD6 knockdown HEK293T cells coexpressing GFP-GAPD and HA-tagged GK2 (mean ± S.D.; n=3; cells counts>200; *P<0.05). Scale bar: 10 μm.

Supplementary Figure S8. Gykl1 and Gk2 regulate mitochondrial ATP level (A) ATP levels in wild-type and *Gykl1* or *Gk2* KO spermatozoa(mean ± S.D.; n=3 independent experiments; **P<0.01, ***P<0.001). (**B**) ATP levels in control, Gykl1, Gk2 or GK2 expressing HEK293T Cells(mean ± S.D.; n=3 independent experiments; *P<0.05, **P<0.01, ***P<0.001.

Supplementary Figure S1

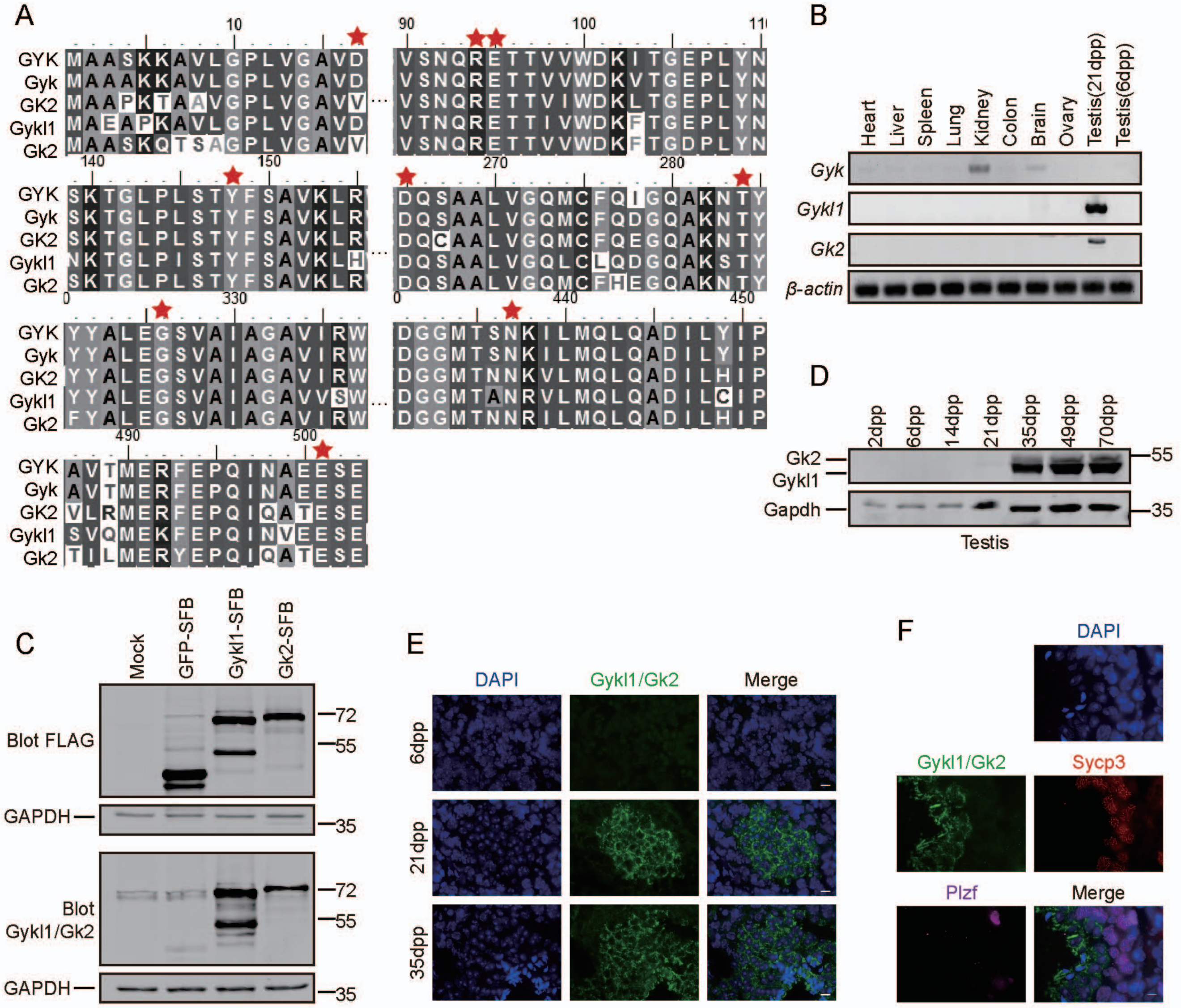

Supplementary Figure S2

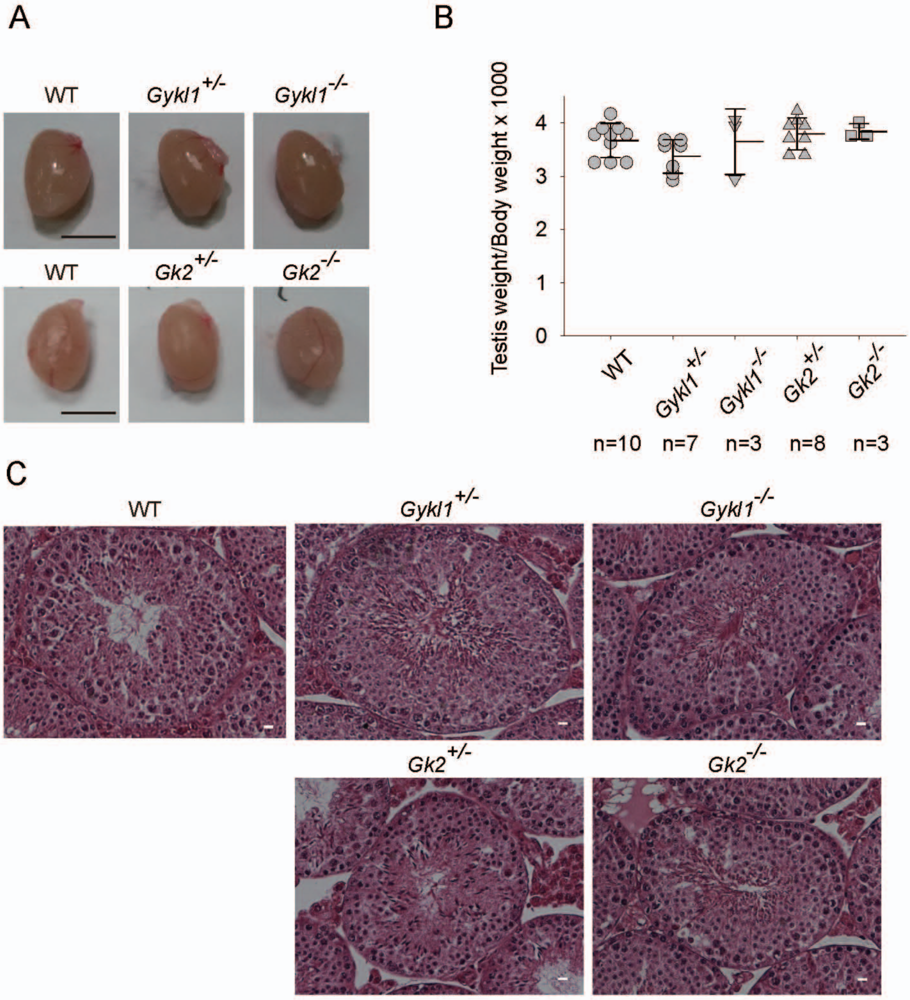

Supplementary Figure S3

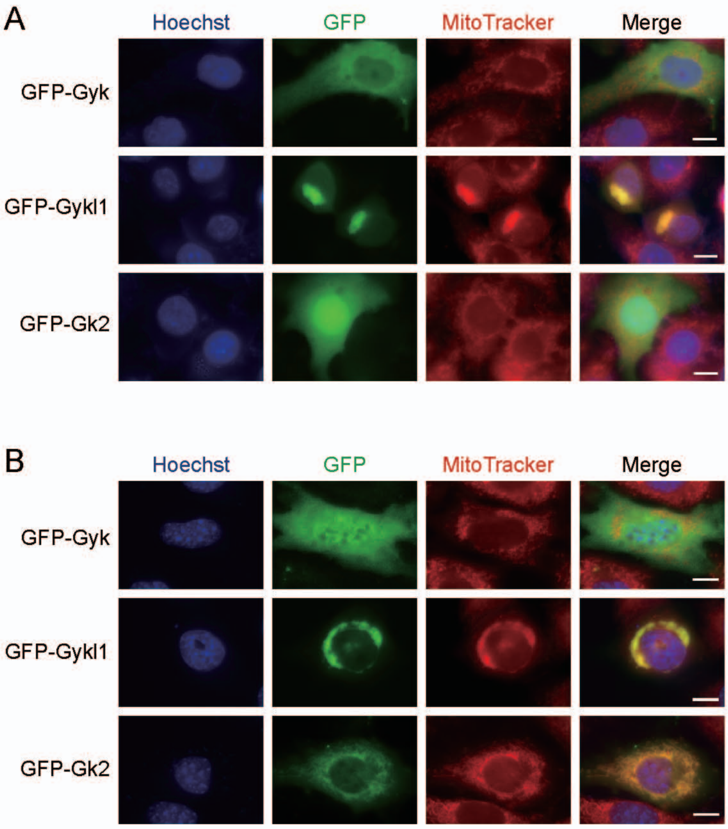

Supplementary Figure S4

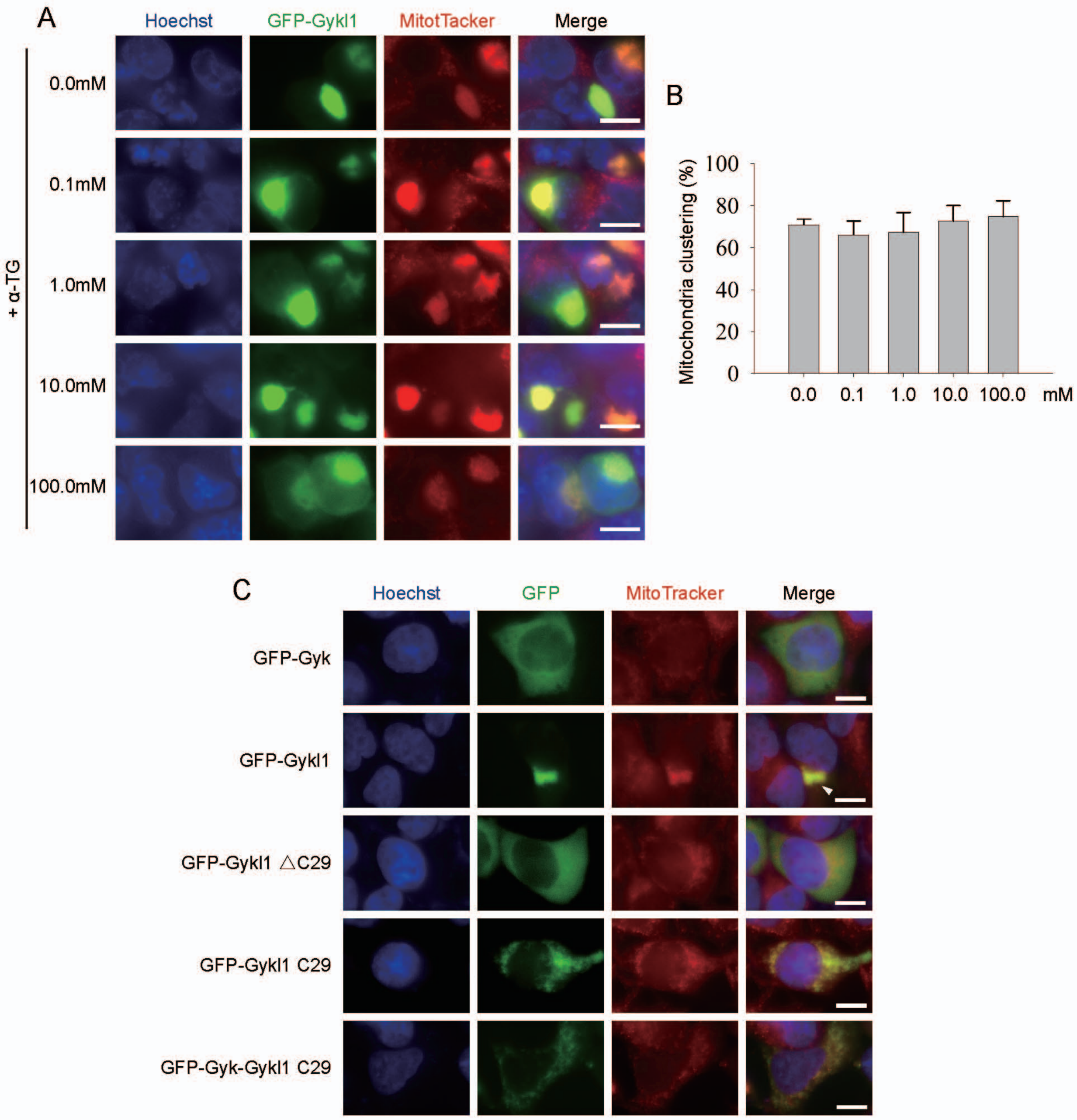

Supplemental Fig.S5

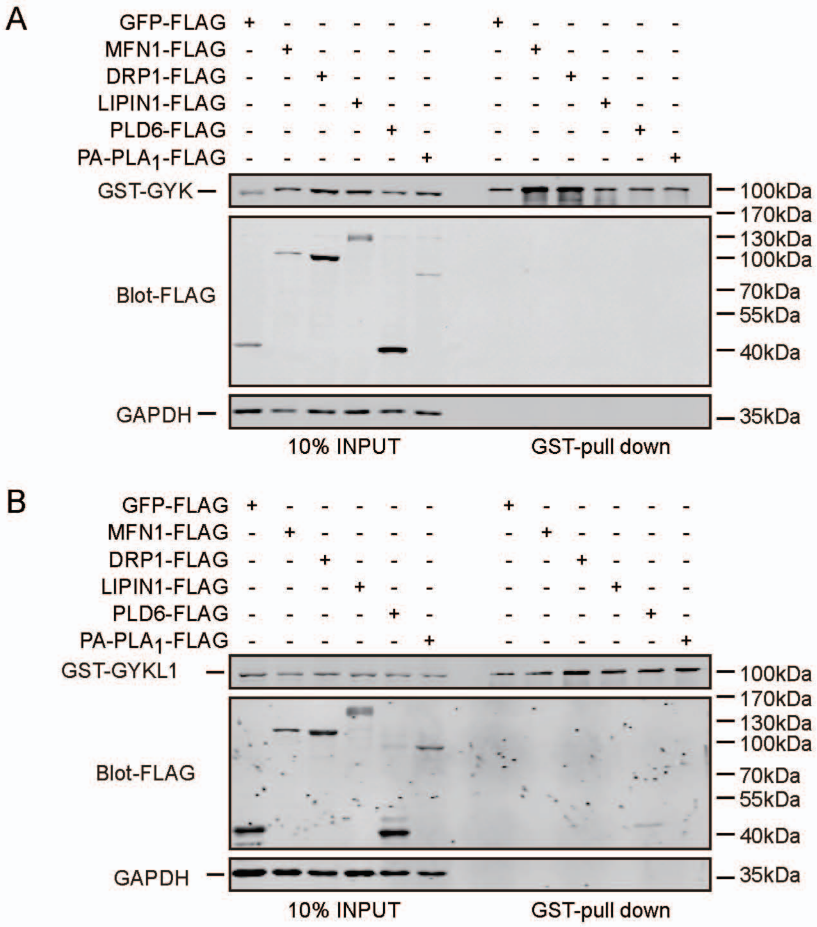

Supplementary Figure S6

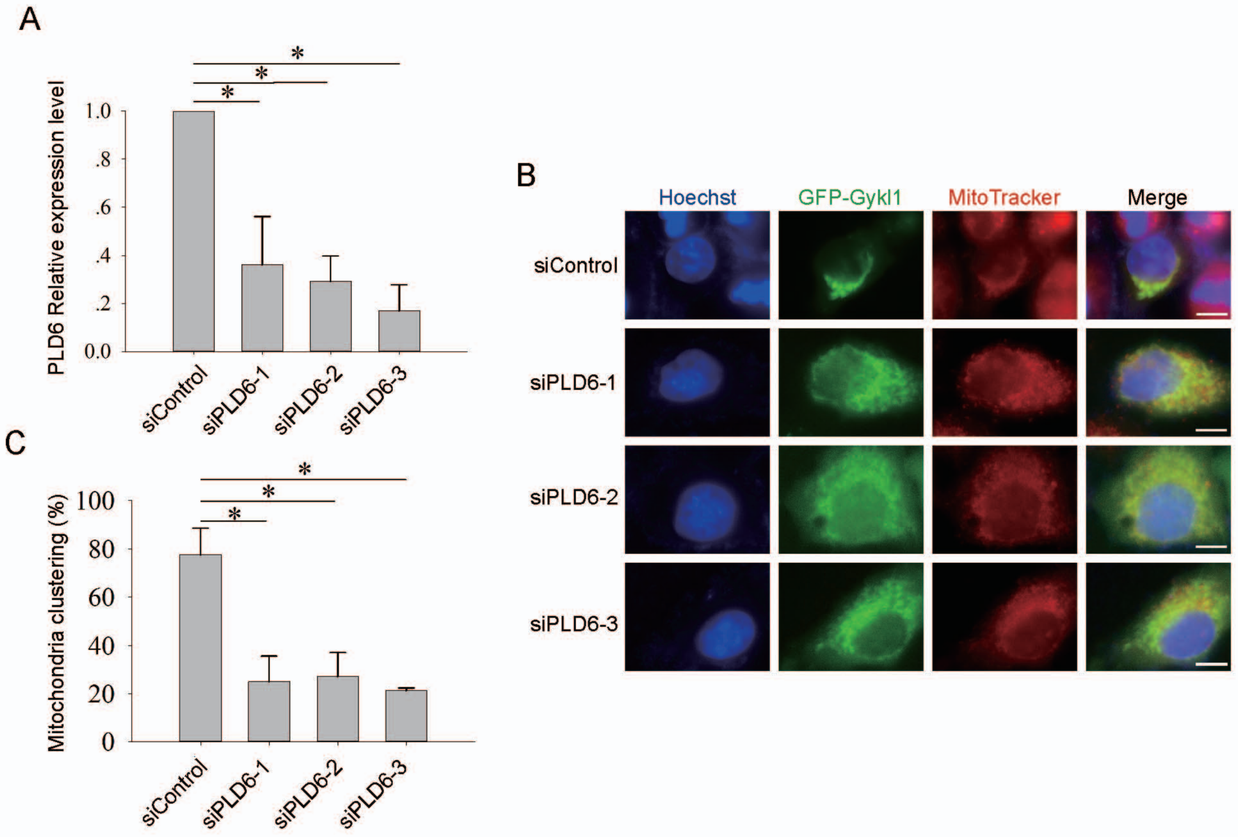

Supplementary Figure S7

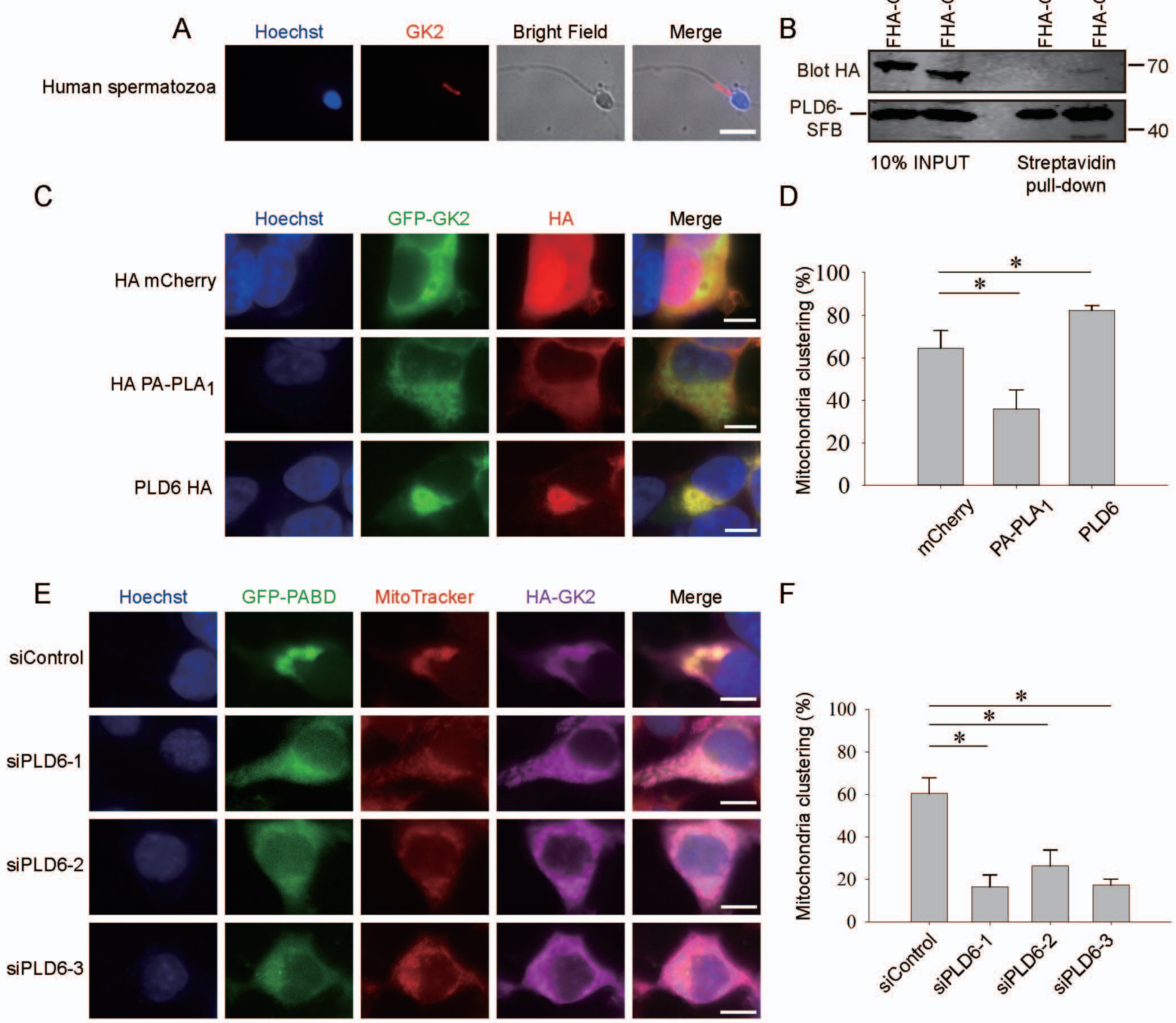

Supplementary Figure S8

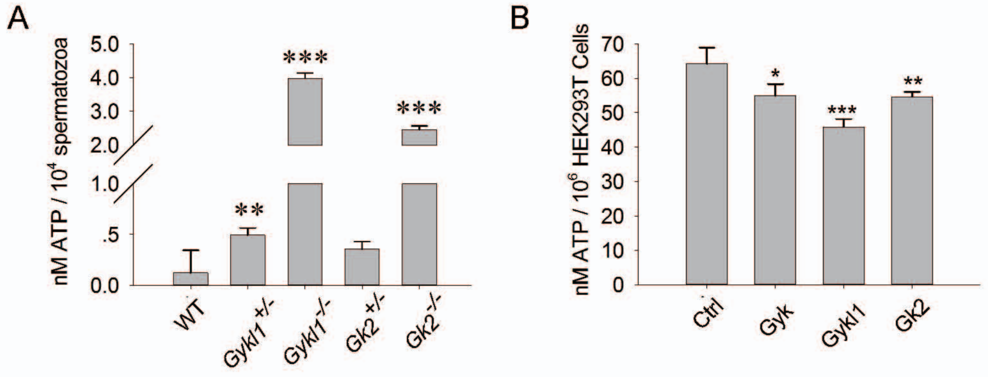

Supplementary Table S1

Intracytoplasmic spermatozoa injection of Gykl1 or Gk2 spermatozoa

Gene	Transplanted embryo	Newborn
Gykl1	12	3
Gk2	47	4*

*Two of Gk2 mice were eaten by the foster mother after birth.
